# The role of body–object interaction in children’s concept processing: insights from two Chinese communities

**DOI:** 10.1007/s10339-024-01185-1

**Published:** 2024-04-08

**Authors:** Zhengye Xu, Duo Liu

**Affiliations:** Department of Special Education and Counselling, The Education University of Hong Kong, NO.10 Lo Ping Road, Tai Po, N.T. Hong Kong

**Keywords:** Community difference, Concept processing, Sensorimotor representations, Word recognition

## Abstract

A rating of body–object interactions (BOIs) reflects the ease with which a human body can interact physically with a word’s referent. Studies with adults have demonstrated a facilitating BOI effect in language tasks, with faster and more accurate responses for high BOI words (e.g., cup) than low BOI words (e.g., coal). A few studies have explored the BOI effect in children. However, these studies have all adopted adult-rated BOIs, which may differ from children’s. Using child-rated BOIs, the present study investigated the BOI effect in Chinese children and its relationship with age, as well as whether there was a community difference in the BOI effect. Children (aged 7–8) from Mainland China (*N* = 100) and Hong Kong SAR (HK; *N* = 90) completed a lexical decision task used to measure the BOI effect. The children were asked to judge whether each item was a real Chinese word; each real word was assigned a child-rated BOI score. After controlling nonverbal intelligence, gender, working memory, and Chinese character reading, a significant BOI effect was observed at the response accuracy and speed levels. The accuracy and latency analyses illustrated a community difference; the BOI effect was smaller in the HK children. This study suggests that BOI measures may be sensitive to the ecological differences between tested communities. The findings support the need for further investigations into the BOI effect across Chinese communities, particularly those in Mainland China.

## Introduction

From the perspective of embodied cognition, previous research has lent support for the role of sensorimotor experiences in lexical processing (Barsalou 2008; Louwerse 2018). One index for the degree of sensorimotor experiences is a rating of body–object interactions (BOIs), reflecting the ease with which a human body can interact physically with a word’s referent (Siakaluk et al. 2008a, b). In alphabetic languages, a significant BOI effect in lexical processing has been reported repeatedly in studies with adult participants (Siakaluk et al. 2008a, b; Tillotson et al. 2008); lexical processing is facilitated if words have high BOI ratings (e.g., cup) more than if they have low ratings (e.g., coal). A few studies (i.e., Inkster et al. 2016; Wellsby and Pexman 2014; Winter et al. 2021) explored the BOI effect in children; however, they adopted adult-rated BOIs. BOIs capture individuals’ experiences of physically interacting with objects, which could be influenced by their daily routines and their bodies. Given the differences between children and adults in daily routines (e.g., children generally have more interactions with items at school than adults do in their daily lives) and bodies (e.g., size), BOI ratings of words from adults might not be appropriate for reflecting ratings from children (Muraki et al. 2022). Therefore, there has been a need for child-rated BOIs, which can yield the BOI effect in children in a direct way.

Furthermore, there is little empirical evidence about the BOI effect in the Chinese language (Xue et al. 2015), in which body parts are critical for expressing concepts (Yu 2009). Moreover, there are many differences between Chinese communities in their approaches to learning Chinese. For example, pinyin, a phonemic coding approach facilitating the acquisition of words’ sounds, is taught in Mainland China (the Mainland) but not in the Hong Kong Special Administrative Region (HK). It has been shown that Chinese-speaking children from the Mainland and HK have dissimilar performances in lexical processing tasks, which some authors have suggested may be related to their differences in learning Chinese (Chen and Yuen 1991; McBride-Chang et al. 2004). However, these previous studies have focused mainly on children’s performances of linguistic-related aspects, e.g., phonological awareness and orthographic knowledge. It is unclear whether the community difference would be present from the perspective of embodied cognition (e.g., the BOI effect). Therefore, with child-rated BOIs, the present study investigated the BOI effect in young Chinese children from two communities (i.e., Mainland and HK) that differ in many aspects of learning. The role of age in the BOI effect was considered for the purpose of exploring the underlying mechanism further.

### BOI effect in lexical processing in alphabetic languages

The BOI effect in lexical processing reflects that conceptual knowledge can be acquired partly from bodily interactions with real-world objects (Barsalou 2008). Physical interactions with objects, e.g., apples, afford more sensorimotor experiences, which can generate and update representations of corresponding concepts, e.g., apple in various sensorimotor modalities such as visual, motor, and taste systems (Louwerse 2018). The more sensorimotor representations lead to richer semantic representations (Barsalou 2008), which can facilitate activations of related orthographic and phonological units (Siakaluk et al. 2008a, b). As a result, lexical processing performance could be improved with higher response speed and accuracy rates.

Few studies have investigated the effect of BOI on lexical processing in children who speak alphabetic languages (i.e., Inkster et al. 2016; Suggate and Stoeger 2014; 2017; Wellsby and Pexman 2014; Winter et al. 2021), but the findings have been mixed. Some studies, consistent with findings from adults (Siakaluk et al. 2008a, b; Tillotson et al. 2008), revealed a significant BOI effect in children (e.g., Inkster et al. 2016; Wellsby and Pexman 2014). Two studies of preschool children (Suggate and Stoeger 2014, 2017) demonstrated a positive correlation between the processing advantage of words with high BOI ratings and fine motor skills. Fine motor skills refer to small muscle movements involving the manual extremities and are manifested through manipulating objects, in particular with the help of fingers and hands (Suggate and Stoeger 2014). Therefore, the correlation between the BOI effect and fine motor skills, at least in part, supported the idea that the facilitating BOI effect is elicited by the involvement of sensorimotor modalities during concept acquisition. In addition to fingers and hands, bodily interactions with objects include other body parts, such as hands, arms, and feet. Hence, to explore the BOI effect further, Winter et al. (2021) extended previous studies by involving body-parts associations with concepts. However, different from the findings reported by Suggate and Stoeger (2014, 2017), Winter et al. (2021) found that when fine motor skills, body-parts associations, and their interactions were considered, the BOI effect was not significant in children, even though it was in adults.

These mixed findings might be related to the BOI ratings of words used in these studies (i.e., Inkster et al. 2016; Suggate and Stoeger 2014, 2017; Wellsby and Pexman 2014; Winter et al. 2021), which were given by adults rather than children. Inkster et al. (2016) utilized parent-reported BOI ratings, while the others adopted ratings from adults who were independent of the participating children. Children and adults have different daily routines, which may lead to different BOI ratings for the same concepts. For example, compared to children, adults may give lower BOI ratings for concepts such as blackboard and textbook; as generally, they interact less with items or objects used in teaching or classroom situations than children do. On the other hand, due to differences in bodies (e.g., size), children and adults could have distinctive experiences when interacting with the same object. For example, when in the same car, adults and children could have different experiences due to their differences in size. Because of their small body sizes, car safety seats are required for young children, which could lead to an experience in a car that adults cannot have. Also, adults could identify more properties of a car through driving, which is not a common experience for children. Thus, using adult-rated BOIs of words makes it difficult to figure out the BOI effect in children directly. Further, it is not clear whether adults' enhanced processing capabilities, e.g., perceptual or linguistic abilities, would modulate the BOI ratings in certain ways. Therefore, collecting direct child-rated BOIs would enhance the validity of comparative studies strongly (Xu and Liu 2022). This study also went a step further to investigate whether BOI ratings were age dependent.

### Similarities and differences between the two Chinese communities

One study of Chinese children from Mainland China revealed a significant BOI effect with a lexical decision task (Xu and Liu 2022). In the study of Xu and Liu (2022), the children recognized the words with higher BOI ratings at higher accuracy rates and higher response speeds. The results suggest that Chinese children can acquire conceptual knowledge through physical interactions with referents (Xu and Liu 2022). In addition to concrete concepts, body-part terms are commonly used to express abstract concepts in the Chinese language (Yu 2009). For example, the concept眉/mei2/ (eyebrow) is incorporated in words 眉飞色舞/mei2-fei1-se4-wu3/(eyebrow-fly-color-dance) and 愁眉不展/chou2-mei2-bu4-zhan3/(worry-eyebrow-no-unfold) to indicate exultant and frowning expressions, respectively. Concepts relating to taste experiences, i.e., 辛/xin1/(spice) and 苦/ku3/(bitter), are used to express laboriousness in concepts such as 辛苦/xin1 ku3/(exhausting). Although the current study focused on concrete concepts, the critical role of body parts in abstract concepts gives rise to a strong connection between bodily interactions and the meanings of concepts.

However, profiles of the reading performances of Chinese-speaking children from different communities are not always consistent. One study found that Mainland children performed better than HK children in phonological awareness tasks (e.g., McBride-Chang et al. 2004). However, reversed results were demonstrated in orthographic processing tasks (e.g., Chen and Yuen 1991). It has been suggested that these community differences in reading may be related to distinctive learning experiences. Specifically, the phonological awareness advantages that Mainland children have over HK children may occur because children learn a phonemic coding approach (i.e., pinyin) in Mainland China. Instead, a look-and-say method is typically used to learn to read characters in HK (McBride-Chang et al. 2004). The difference in the orthographic processing is attributed to the script used in HK (i.e., traditional Chinese) having approximately 22.5% more strokes than the simplified Chinese used in Mainland China (Gao and Kao 2002).

Notably, differences between these two communities are not limited to the linguistic-related aspects but include aspects relating to the BOI effect, that is, bodily interactions with objects. These differences give rise to two possibilities about the community difference in terms of the BOI effect. On the one hand, HK children have many after-school private tutorials in academic subjects (Bray 2013), leading to a greater emphasis on indoor activities. Some indoor activities, such as handwriting, are primarily associated with fine motor skills, which have been linked to the BOI effect (Suggate and Stoeger 2014, 2017). Hence, the first possibility regarding the BOI effect between the two communities is that HK children may exhibit larger BOI effects.

On the other hand, due to a higher population density in HK (i.e., 6890/km^2^; Census and Statistics Department 2021) compared to the Mainland (i.e., 580/km^2^ of Huzhou, the city from which the current sample was taken; Zhejiang Provincial Bureau of Statistics 2021), HK children have smaller spaces for activities in their homes, schools, or communities. At the same time, HK children have less time for outside activities, as physical education lessons for primary school students are shorter in HK (i.e., about 80 to 120 minutes per week; Education Bureau 2017) than in Mainland China (i.e., about 160 to 180 minutes per week, Ministry of Education of the People’s Republic of China 2011). Physical activity is positively correlated to children’s motor development, including both gross and fine motor skills (e.g., Dapp et al. 2021; Sääkslahti and Niemistö, 2021), which are associated with object manipulation (Winter et al. 2021). Therefore, another possibility is that the relatively smaller spaces and fewer opportunities for physical interaction with the external environment, particularly large dynamic spaces, may result in fewer sensorimotor experiences for HK children. Consequently, it is hypothesized that HK children may exhibit smaller BOI effects in lexical processing compared to children from the Mainland.

### Present study

In sum, to investigate the BOI effect in Chinese children, participants from Huzhou in Zhejiang province, an eastern city of the Mainland, and HK were recruited. They were aged 7–8, the age at which word-recognition behavior begins to be influenced by variables relating to meaning richness (Schwanenflugel and Noyes 1996). The participants were asked to complete a lexical decision task for the BOI effect, followed by a rating task for the BOIs of the real words used in the lexical decision task. Due to their influence on word recognition, nonverbal intelligence, gender, and working memory were controlled for. Given the different scripts and spoken languages in these two communities, the linguistic/verbal stimuli in the instrument described below were presented in simplified Chinese and Putonghua for the Mainland children and in traditional Chinese and Cantonese for the HK children. These differences in stimuli might influence lexical processing. Hence, Chinese character reading, a reading ability at the word level, was measured and statistically controlled (Liu et al. 2017). The study had three research questions: (1) Whether there would be a relationship between BOI and word recognition in Chinese children; (2) whether there would be a community difference in the BOI effect; and (3) whether there would be an age difference in the BOI effect.

## Method

### Participants

In total, 110 Mainland children and 102 HK children were recruited from equivalent mainstream primary schools catering to children from middle-class backgrounds. Written informed parental consent was obtained from the participants’ legal guardians, and verbal assent was received from each child. This study was approved by the [name deleted to maintain the integrity of the review process] according to the Declaration of Helsinki. The schools and parents reported no physical or mental conditions in any of the children. All data from four Mainland children and six HK children were excluded as their overall accuracy on the lexical decision task was lower than 60%. In addition, six Mainland children and six HK children were excluded to ensure that the two community samples were matched for age. The remaining 100 Mainland children (47 boys; Mean age = 7.87, ranging from 7.30 to 8.47) and 90 HK children (43 boys; Mean age = 7.93, ranging from 7.49 to 8.38) were all native speakers of Chinese. The data were compared both with and without the samples matched for age. Overall, as well as by community, there were no significant differences in any of the measures (*p*s > 0.05).

### Procedure

The participants were asked to complete a lexical decision task for the BOI effect. Then, they undertook tasks for nonverbal intelligence, working memory, and Chinese character reading, which were conducted in a counterbalanced order. Finally, they were asked to complete a rating task for the BOI values of the real words in the lexical decision task.

### Measures

#### Lexical decision task

A lexical decision task was adopted from previous studies (Siakaluk et al. 2008a, b). The task consisted of 120 experimental trials. Of these, 60 trials were two-character real words, and the remaining 60 were nonwords. The real words were selected from the commonly used Chinese textbooks for primary schools in each community, while the nonwords were developed by combining characters from the textbooks. Each trial started with presenting a fixation cross at the center of the screen for 400 ms. Next, a real word or a nonword appeared at the center of the screen, and the participant was asked to judge, as quickly and as accurately as possible, whether the word made sense. Half of the participants were instructed to make a “yes” response by pressing the “F” key on the keyboard or to make a “no” response by pressing the “J” key; the other half of the participants made responses by using the reversed keys. Immediately after the response, or after 2000 ms had passed, a blank screen appeared for 1000 ms. Each participant began with ten practice trials, which differed from the experimental trials and consisted of five real words and five nonwords. The same real words and nonwords for both the practice and experiment sessions were used for the Mainland and HK children. All the trials were presented in a random order, and the task took approximately 10 min to complete.

#### Nonverbal intelligence

Sets A and B of Raven’s Standard Progressive Matrices (Raven 1996) were administered to measure the children’s nonverbal intelligence. For each test item, the children were asked to select from six options to complete an image with a missing element. One point was given for each correct answer, and the maximum score was 24. The Cronbach’s alphas were 0.85 and 0.78 for the participants in the Mainland and HK, respectively.

#### Working memory

In a backward digit recall task (Alloway and Alloway 2010), the children were asked to recall a sequence of spoken digits, varying from two to eight strings, in reverse order. There were 14 sequences, and the maximum score was 14. The Cronbach’s alphas were 0.77 and 0.89 for the children in the Mainland and HK, respectively.

#### Chinese character reading

In HK, a Chinese character reading test (Liu et al. 2017) was utilized to examine the children’s reading abilities at the word level. The task consisted of three character lists specific to Grades 1, 2, and 3 in primary school. Each list contained 72 characters, and one point was given for each correct answer. Each list stopped when a child failed to recognize 15 consecutive characters. In the Mainland, a test of character reading (Liu and McBride-Chang 2010), similar to that used in HK, was adopted. However, it was shortened and modified; there were 100 items. The Cronbach’s alphas were 0.98 and 0.94 for the participants in the Mainland and HK, respectively.

#### BOI rating

The BOI rating procedure was the same as the lexical decision task, except that no nonwords were presented. Instead of judging whether words made sense, the children were asked to rate on a 1–7 scale how easily they could interact physically with each word’s referent (1 = extremely easy; 7 = extremely hard). The participants were asked to give two examples of words, one with a relatively high BOI rating (e.g., pencil) and one with a low (e.g., coal) rating, before they did the actual rating, to confirm that they understood the instructions. The Cronbach’s alphas were 0.95 and 0.96 for the participants in the Mainland and HK, respectively.

### Data analysis

Since the lexical decision task was utilized to measure the BOI effect of word recognition, only the 60 real words were considered in the analysis. Using the *lmer* program of the lme4 package in R 4.0.3 (R Core Team 2020), accuracy was analyzed in a generalized mixed-effects logistic model for binomial data, and response time (RT) data were examined in a linear mixed-effects model. For the RT analysis, incorrect responses were removed. RTs that were three standard deviations above or below a participant’s condition mean for correct responses (6.85% for the Mainland and 3.34% for HK) were excluded (Marmolejo-Ramos et al. 2015).

The same predictors were used for the accuracy and the RT models. The BOI rating, age, community, and the 2-way and 3-way interactions between them were entered in the models as independent variables. For each community, the BOI rating of each word was the average of all participants in the community. Nonverbal intelligence, gender, working memory, Chinese character reading, and the frequencies (mean frequency was 0.04, ranging from 0.005 to 0.16; Da 2004) and complexities (i.e., the sum of strokes of the two characters in the word) of the real words were entered into the models as covariates. Chinese character reading was transformed into *z* scores by the community before entering the models. The average complexities were 15.37 (ranging from 6 to 27) in the task for the Mainland children and 18.77 (ranging from 7 to 38) for the HK children. Random intercepts by participants and items were entered first. Next, random slopes for significant fixed factors of interest were forward-fitted unless adding one could lead to the model failing to converge (Meteyard and Davies 2020). A more complex model is considered acceptable if there is a better goodness of fit, i.e., a difference of more than 2 in the Akaike information criterion (Baayen et al. 2008). RT, the BOI rating, age, nonverbal intelligence, working memory, and the complexity and frequency of the real words were centered by the *z*-score transformation of raw scores to overcome the problem of collinearity. The data and analysis code are available from https://osf.io/h9ew4/.

## Results

The descriptive statistics for all measures are summarized in Table 1. Because the tasks were derived indigenously in each community and thus were not parallel, comparisons of raw scores between communities were not conducted. The results of the models (see Table 2) showed significant BOI effects; the accuracy rate and the response speed of the lexical decision task were increased with the BOI rating of the word. The interaction between the BOI rating and the community was significant in both the accuracy and latency analyses, with the BOI effect smaller for the HK children than for the Mainland children.

**Table 1:** Descriptive statistics for children from Mainland China (Mainland) and Hong Kong Special administrative region (HK)

	Mainland (*N* = 100)		HK (*N* = 90)	
	M (SD)	Range	M (SD)	Range
Accuracy	0.89 (0.10)	0.60–1.00	0.88 (0.09)	0.62–0.98
Response time (ms)	1160.26 (221.23)	665.05–1723.02	1145.83 (113.14)	913.02–1374.93
Nonverbal intelligence	19.37 (2.55)	11–24	17.66 (3.38)	9–23
Working memory	4.90 (1.72)	2–11	4.83 (2.38)	1–13
Chinese character reading	71.62 (10.89)	44–92	160.52 (25.31)	88–199
BOI rating	4.45 (0.66)	3.33–5.58	4.42 (0.94)	2.50–6.16

Accuracy and response time are the results of the lexical decision task; BOI = body–object interaction. The Chinese character reading task for the children from the two communities was similar, but the maximum scores of the task used in the Mainland and HK were 100 and 216, respectively

**Table 2:** Mixed-effects models predicting children’s response accuracy and latency in the lexical decision task

Model equation	Accuracy/response time ~ BOI * Community * Age + Nonverbal intelligence + Gender + Working memory + Reading + Complexity + Frequency + (1 + BOI + Frequency |Participant) + (1 + Reading |Stimuli)							
	Accuracy				Response time			
Fixed Effects	Estimate	*SE*	*t*	*p*	Estimate	*SE*	*t*	*p*
BOI	0.18	0.06	2.88	0.004	− 0.15	0.02	− 6.27	< 0.001
Community	− 0.27	0.16	− 1.68	0.094	0.03	0.06	0.46	0.647
Age	0.01	0.12	0.10	0.920	− 0.05	0.04	− 1.13	0.260
Nonverbal intelligence	− 0.02	0.08	− 0.21	0.834	0.01	0.03	0.24	0.812
Gender	0.09	0.15	0.61	0.540	0.10	0.05	1.95	0.052
Working memory	− 0.05	0.07	− 0.66	0.512	− 0.01	0.03	− 0.40	0.691
Reading	0.10	0.08	1.22	0.223	− 0.08	0.03	− 2.72	0.007
Complexity	0.03	0.05	0.69	0.493	0.03	0.02	1.85	.066
Frequency	0.27	0.06	4.30	< 0.001	− 0.08	0.02	− 4.21	< 0.001
BOI × Community	− 0.20	0.06	− 3.14	0.002	0.10	0.02	4.53	< 0.001
BOI × Age	0.08	0.05	1.79	0.073	− 0.03	0.02	− 1.88	0.060
Community × Age	− 0.29	0.19	− 1.58	0.113	0.05	0.07	0.75	0.454
BOI × Community × Age	− 0.06	0.07	− 0.84	0.400	0.03	0.03	1.14	0.256
Random effects	Variance	*SD*			Variance	*SD*		
Participant (intercept)	0.89	0.94			0.13	0.36		
BOI by participant (slope)	0.001	0.04			0.005	0.07		
Frequency by participant (slope)	0.03	0.17			0.002	0.04		
Stimuli (intercept)	0.08	0.29			0.02	0.14		
Reading by stimuli (slope)	–	–			0.001	0.03		

BOI = body–object interaction; Location = the contrast between Mainland China and Hong Kong SAR, with Mainland China as the reference. Gender = the contrast between the boys and girls (reference); Reading = Chinese character reading; Complexity and Frequency are the complexity and frequency of the real words, respectively, in the lexical decision task. The random slope for Reading was not entered in the response time model due to the failure of converge

The effect of one control variable, the frequency, was significant in both models. The words with higher frequencies led to higher accuracy and shorter RTs. The effect of Chinese character reading was significant in the latency analysis. The children with higher scores on the Chinese character reading task gave correct responses more quickly.

## Discussion

The current study compared the strengths of the BOI effect, i.e., the association between the child-rated BOI and word recognition, in Chinese children from the Mainland and HK. The age effect on the association was examined as well. The BOI effect and its interaction with age were significant in both the accuracy and latency analyses. More importantly, the significant community difference in the BOI effect was observed in the latency analysis.

### BOI effect in Chinese children

With the child-rated BOIs, the current study illustrated the significant BOI effect on both the response accuracy and speed levels in Chinese children. However, the previous studies in alphabetic-language-speaking children (i.e., Inkster et al. 2016; Wellsby and Pexman 2014) only observed the BOI effect in the latency analysis. One interpretation is that the current study utilized the child-rated BOI, which enabled the association between the children’s bodily experiences with objects and word recognition to be investigated more appropriately. This interpretation is consistent with findings in adults; with BOI ratings from individuals in the same age group as the participants (i.e., adults), the BOI effect was significant at the response accuracy and speed levels (e.g., Siakaluk et al. 2008a, b). Alternatively, the BOI effect on the accuracy level might have been induced partly by the feature that body-part terms and related events are commonly used to express Chinese concepts (Yu 2009). This feature might highlight the role of sensorimotor experiences in acquiring and processing concepts in the Chinese language, resulting in benefits at the response speed and accuracy levels (Li et al. 2007). At the same time, although the current sample only involved a narrow age range, the results demonstrated that the BOI effect increased with age. It is possible that the BOI effect, as an aspect of cognition, is sensitive to age, as older children tend to demonstrate higher accuracy rates and faster processing speed in language tasks.

The overall performance on the lexical decision task was similar for the children from the two communities; however, the accuracy and latency analyses showed significant community differences in the BOI effect. The HK children had smaller BOI effects than those of the Mainland children. Such a community difference may have been elicited by fewer sensorimotor representations in the mental lexicon of the HK children relative to those of the Mainland children. As mentioned earlier, HK children, in general, tend to have less time for regular or nonregular physical activities, particularly in large dynamic environments, compared to their Mainland counterparts. It is possible that interactions in dynamic outdoor spaces may influence the BOI effect, and this aspect should be further explored in future research.

In addition, this community difference in the BOI effect suggests that children’s BOI ratings are sensitive to various aspects. These aspects might include linguistic, physiological, perceptual, cultural, and ecological differences between communities, all of which might influence the role of bodily interactions with objects in conceptual processing (Barsalou 2008; Wellsby and Pexman 2014). For example, it might be related to differences in children’s linguistic skills. Since Chinese is the first language of all children from the two Chinese communities in the current sample and their reading ability (i.e., Chinese character reading) was controlled, the present study did not further explore the influence of the participants’ linguistic skills in other languages. However, many children in Hong Kong become proficient Chinese (L1)–English (L2) bilinguals from an early age (Wang et al. 2010), and literature on bilinguals has also shown the effect of the learning experience of L2 on children’s literacy performance in L1 (e.g., Chen et al. 2010; Choi et al. 2018). For example, Chen et al. (2010) found that English instruction can facilitate children’s phonological awareness and pinyin (i.e., an alphabetic script with regular letter-sound correspondence) skills. In the context of embodied cognition, some studies have found a larger extent of involvement of sensorimotor representations in processing the first language than the second language (e.g., Vukovic and Shtyrov 2014). It has been suggested that this difference between L1 and L2 may be due to the rich sensorimotor contexts in which early L1 acquisition occurs (e.g., Vukovic and Shtyrov 2014). The influence of the second language on the first language in terms of the BOI effect has not been explored yet. However, it is possible that similar to the situation in literacy performance (e.g., Chen et al. 2010; Choi et al. 2018), children’s L2 (e.g., English) learning experiences can lead to differences in L1 (e.g., Chinese) in relation to sensorimotor representations.

The community difference in the BOI effect might also be related to other environmental factors, such as socioeconomic status (SES, e.g., parental incomes and educational levels). SES has been found to play a critical role in children’s linguistic achievements (Pinquart and Ebeling 2020; Tan et al. 2020). A possible explanation for the differences in BOI is that variants in socioeconomic backgrounds may lead to unequal access to resources and opportunities for physical interactions with objects that impact BOI experiences and understanding. Previous studies have demonstrated a negative correlation between SES and children’s daily screen time (e.g., Tandon et al. 2012), which has been found to be negatively correlated with the extent of the BOI effect (Xu and Liu 2022).

In addition, children’s characteristics, especially those associated with their interactions with objects, may result in differences in the BOI effect. To illustrate, two aspects of grit, i.e., consistency of interest and perseverance of effort, can influence children’s engagement during tasks (Karlen et al. 2019). Previous studies have found that children with higher levels of grit tend to perform better academically (Cosgrove et al. 2018). In addition to formal learning activities, these two aspects might also affect children’s physical interactions with the objects around them, leading to variances in the extent of the BOI effect. As well as being a critical factor influencing learning (Ainley et al. 2002), children’s interests might impact their building of sensorimotor representations. A child interested in exploring objects with physical interactions is likely to be involved in these activities more frequently and to participate in them more deeply. More importantly, these diverse factors may interplay to influence the BOI effect in children, which should be investigated further in the future.

### Limitations, future studies, and conclusions

As one of the first studies investigating the BOI effect in Chinese children, the present study controlled the possible impacts of latent variables to some extent by controlling Chinese character reading, which has correlations with latent variables such as socioeconomic status (Liu et al. 2016) and daily routines (Trudeau and Shephard 2008). Future studies could take these latent variables directly into account. To figure out possible reasons leading to the community differences in the BOI effect, children in different communities within Mainland China should be compared. Their physical activities, as well as their fine and gross motor skills, which are related to physical activities and could affect physical interactions with referents (Suggate and Stoeger 2014, 2017; Winter et al. 2021), could be measured and compared between communities in future studies. At the same time, there is a need for further examination of the influences of more factors from diverse aspects (e.g., linguistic and ecological differences). Also, longitudinal studies can be conducted to identify a complete developmental pattern. In addition, Winter et al. (2021) suggested a possible difference in the BOI effect between children and adults, which was not investigated in the current study. Future studies could involve participants from different age groups, i.e., young children, older children, and adults, with BOI ratings from the corresponding participants or those in the same age groups as the participants, to further investigate the underlying mechanism of the BOI effect.

The current study extends previous findings with the child-rated BOI by demonstrating the BOI effect in Chinese children at accuracy and latency levels. Moreover, from the current results showing the community difference in the BOI effect, a possibility emerges that the embodied system could have different levels of involvement during lexical processing in children with various daily routines. Practically, the current findings suggest that bodily interactions with objects, even without explicit teaching and learning, could form a basis for developing conceptual knowledge. More leisure activities, which could assist children to interact with different objects in dynamic environments, should be involved in their daily lives.

## Data Availability

The data supporting this study's findings are available from https://osf.io/h9ew4/.
